# Molecular Targets in Lung Cancer: Study of the Evolution of Biomarkers Associated with Treatment with Tyrosine Kinase Inhibitors—Has NF1 Tumor Suppressor a Key Role in Acquired Resistance?

**DOI:** 10.3390/cancers14143323

**Published:** 2022-07-07

**Authors:** Begoña O. Alen, Lara S. Estévez-Pérez, María Teresa Hermida-Romero, Ana Reguera-Arias, Rosario García-Campelo, Mercedes de la Torre-Bravos, Ángel Concha

**Affiliations:** 1 Department of Anatomical Pathology, University Hospital Complex A Coruña, 15006 A Coruña, Spain; teresa.hermida.romero@sergas.es (M.T.H.-R.); ana.reguera.arias@sergas.es (A.R.-A.); angel.concha.lopez@sergas.es (Á.C.); 2 Molecular Biology Area, Department of Anatomical Pathology, University Hospital Complex A Coruña, 15006 A Coruña, Spain; 3 Department of Oncology, University Hospital Complex A Coruña, 15006 A Coruña, Spain; ma.rosario.garcia.campelo@sergas.es; 4 Department of Thoracic Surgery, University Hospital Complex A Coruña, 15006 A Coruña, Spain; mercedes.de.la.torre.bravos@sergas.es; 5 Biobank of A Coruña, Instituto de Investigación Biomédica A Coruña (INIBIC), 15006 A Coruña, Spain

**Keywords:** lung cancer, EGFR, tyrosine kinase inhibitors, liquid biopsy, NGS, NF1

## Abstract

**Simple Summary:**

Resistance to tyrosine kinase inhibitors in patients with EGFR-mutated non-small cell lung cancer is crucial in the development of the disease. Detecting the mechanisms of this resistance is fundamental in lung cancer research, so we evaluated the presence of EGFR mutations in circulating free DNA in plasma of patients with NSCLC under oncological treatment. We studied the role of EGFR and other driver mutations in their involvement in acquired resistance to treatment with EGFR-TKIs and we analyzed the role of liquid biopsy as a non-invasive diagnostic method. Our results showed that liquid biopsy is a very useful tool monitoring the evolution of the disease and the resistance to TKIs. The detection of other concomitant mutations in driver genes is also key in this regard, so we found that alterations in the NFI tumor suppressor gene could be playing a role in disease progression and resistance to targeted therapies.

**Abstract:**

The application to clinical practice of liquid biopsy in patients with lung cancer has led to an advance in the diagnosis and monitoring of the disease. Detection of alterations in EGFR genes related to TKI treatment in EGFR-mutated non-small cell lung cancer patients is a routine method in pathology laboratories. The primary objective of this work was to analyze the presence of EGFR mutations in cfDNA of 86 patients with lung cancer undergoing oncological treatment related to response to treatment with TKIs. Secondarily, we evaluated the dynamics of EGFR mutations, the presence of the T790M alteration and its relationship with drug resistance and analyzed by NGS molecular alterations in cfDNA of patients with discordant progression. Our results demonstrate that understanding the mutational status of patients treated with TKIs over time is essential to monitor disease progression. In this context, liquid biopsy is a fundamental key. In addition, it is not only necessary to detect EGFR mutations, but also other concomitant mutations that would be influencing the development of the disease. In this sense, we have discovered that mutations in the NF1 tumor suppressor gene could be exerting an as yet unknown function in lung cancer.

## 1. Introduction

In recent years, personalized therapeutic strategies based on target-specific drugs or immunological treatments have been developed with very encouraging results. These treatments could become neoplastic disease in chronic, extending the overall survival (OS) and the disease-free survival (DFS) and improving the quality of life of patients [1,2]. One of these strategies consists in the administration of drugs that are inhibitors of the tyrosine kinase activity (TKIs) of the growth factor receptors involved in carcinogenesis [3]. This is the case of the epidermal growth factor receptor (EGFR) in patients with non-small cell lung carcinoma (NSCLC) [1] harboring EGFR mutations in exons 18, 19, 20 and 21 [4]. The determination of the presence of these sensitive mutations is crucial to indicate TKI treatment, therefore, routine EGFR mutation determinations in diagnosis became indispensable [5,6]. In addition to the natural primary resistance to these drugs (intrinsic resistance), acquired resistance after their administration occurs throughout the treatment. For this reason, first, second, third and shortly fourth generation TKIs [7] such as osimertinib or rociletinib have been obtained to overcome this drawback. The mechanisms of emergence of acquired resistance are multiple and not all well known, but it is the acquisition of the T790M mutation [8,9,10], in exon 20 of EGFR, that is the main cause identified to date. Therefore, it is very important to know if patients with NSCLC undergoing treatment with TKIs, and who suffer disease progression, have acquired the T790M mutation in their neoplastic cells to decide if they should receive treatment adjusted with third generation TKIs.

Liquid biopsies allow obtaining molecular information of the neoplastic process at each moment of the patient’s evolution, including as a method of screening/early detection [11], monitoring responses to treatments, detecting minimal persistent residual disease or early recurrence before it manifests clinically. They can also be used to determine the molecular profile of the tumor at the beginning of the diagnosis if there is no sufficient or available tissue sample and to study new alterations and mutations that may arise during the course of the disease [12,13]. One of the great disadvantages of their application in clinical practice is the lack of standardization of the different procedures currently available and the lack of consensus or recommendations of clinical guidelines endorsed by scientific societies. However, it was assumed that they are a tool with increasing importance in clinical management and should be performed if the patient has an unexpected evolution or progression of the disease. In fact, recently, several clinical guidelines and consensus statements for their application in oncology were published [14,15]. Furthermore, only two procedures with IVD-CE validation and approved by the FDA for clinical use could be used until recently: the CELLSEARCH system (Menarini Silicon Biosystems, Huntingdon Valley, PA, USA) for the detection of circulating tumor cells (CTCs) in the case of breast, colon and prostate cancer; and the cobas^®^ EGFR Mutation Test V2 system (Roche Diagnostics, Mannheim, Germany) for cell-free DNA (cfDNA) in lung cancer. Lately, several single- or pan-cancer tests have been approved by the FDA, including two NGS circulating tumor DNA (ctDNA) liquid biopsy solutions indicated for use with two or more solid cancers [16].

When patients with NSCLC treated with TKIs present disease progression, the presence of T790M in plasma is studied to adapt the treatment if the result is positive [12]. Otherwise, we might be confronting different situations: (1) T790M mutation exists but we are not able to detect it due to analytical precision. (2) T790M mutation is present in primary tumor or at least one of the metastatic foci but the cfDNA is not exported to the bloodstream because cells do not undergo necrosis/apoptosis processes. (3) There are other biological phenomena that explain the evolution of the disease. The existence of the EGFR-C797S or other 3rd generation-resistant mutations [17], c-MET amplification or the transformation of the NSCLC into a small cell lung cancer (SCLC) [18] are already recognized processes, among others, as the cause of this acquired resistance. In addition, other processes of intrinsic resistance that have not been evaluated must be taken into account. In all these cases, it is recommended to perform other types of molecular studies to be able to adjust the specific treatment. Next generation sequencing (NGS) techniques are emerging with great force in the panorama of “precision medicine in oncology”, applied to tissue samples and liquid biopsies. They provide a large amount of complex information on the mutational profile of each tumor, and allow not only to increase the knowledge of the processes involved in the initiation, progression and tumor dissemination, but also to adjust the available treatments specific to the molecular profile of the neoplasm in particular [19,20].

In this context, the primary objective of this work was to analyze the presence of EGFR mutations in cfDNA in plasma of 86 patients with lung cancer undergoing oncological treatment and to study their relationship with the response to treatment with TKIs. Secondarily, we evaluated the dynamics of these EGFR mutations with respect to the primary tumor, the presence of the T790M mutation and its relationship with drug resistance to TKIs and analyzed, by NGS, molecular alterations in the cfDNA of patients with NSCLC undergoing TKI treatment that have unexplained acquired resistance.

## 2. Materials and Methods

### 2.1. Patients and Samples

This research was conducted as a single-center retrospective study between May 2016 and February 2022. The samples were collected and analyzed by the Pathological Anatomy Service at the University Hospital Complex of A Coruña. The Pathological Anatomy Laboratory is UNE-EN ISO 9001-2015 certified. Clinical data such as gender, age, tumor histology, smoking habits, disease stage or cancer treatment were obtained from the medical records. The patients belong to a study approved by the Clinical Research Ethics Committee (approval registration number 2019/358) and it was conducted in compliance with the Declaration of Helsinki. Written informed consents, custody and remnant sample storage were managed by the Biobank of A Coruña. The Biobank of A Coruña, UNE-EN ISO 9001-2015 certified, ensured the traceability and quality of plasma samples for use in the study.

From a cohort of lung cancer patients undergoing EGFR mutations analysis by the Pathological Anatomy Service at the University Hospital Complex of A Coruña, a total of 158 cfDNA plasma samples belonging to 86 NSCLC patients suitable to be treated by TKIs were included in this study. All these patients were the subjects who underwent liquid biopsy in our hospital by medical recommendations. Most patients presented an advanced clinical stage (III–IV) according to the latest AJCC Cancer Staging Manual of the TNM classification for lung cancer [21] at the moment of the analysis and treatment, follow-up and development of the disease were carried out in the University Hospital Complex of A Coruña. All patients underwent analysis of EGFR mutations in plasma and, in most cases, also tissue biopsy for healthcare causes. In cases with discordant progression and clinical evolution who did not respond to treatment and/or in which T790M TKI resistance mutation was not detected, NGS was performed. In addition to EGFR mutations, alterations in ALK and ROS-1 were analyzed in all patients. In subjects diagnosed after 2018, PD-L1 was also studied as recommended by international guidelines [6]. Demographic parameters and baseline characteristics of the patients were recorded and are shown in Table 1.

### 2.2. Extraction of cfDNA from Plasma Samples for EGFR Mutation Analysis

Peripheral whole blood was collected from each subject in a 10 mL EDTA-K2 tube and processed after centrifugation within 4 h to avoid contamination with genomic DNA released from lysed blood cells. Samples were centrifuged at 4000 rpm for 20 min to collect 2 to 4 mL of plasma and stored at −20 °C. Processing of plasma samples and isolation of cfDNA was carried out using the commercial cobas^®^ cfDNA Sample Preparation Kit (Roche Diagnostics, Mannheim, Germany) as per the manufacturer’s instructions.

### 2.3. Molecular Characterization of EGFR in cfDNA

All cfDNA samples (n = 158) were further analyzed by cobas^®^ EGFR Mutation Test v2 (Roche Diagnostics, Mannheim, Germany) following the manufacturer’s recommendations. This assay kit is approved by the FDA for diagnosis, valid for both FFPE and plasma samples and detects 41 mutations in exons 18, 19, 20 and 21 of the EGFR gene. Real-time PCR was performed on the Roche cobas^®^ z 480 analyzer (Roche Diagnostics, Mannheim, Germany).

### 2.4. Extraction of cfDNA from Plasma Samples for NGS Analysis

Cases with discordant progression and clinical evolution who did not respond to treatment and/or in which T790M TKIs resistance mutation was not detected were analyzed by an NGS targeted panel. Four milliliters of plasma was used for cfDNA extraction using a QIAmp Circulating Nucleic Acid kit (QIAGEN, Hilden, Germany), according to the manufacturer’s instructions. The cfDNA was eluted with 30 μL elution buffer and quantified using a Qubit dsDNA HS assay kit (Thermo Fisher Scientific, Waltham, MA, USA).

### 2.5. Validation and Molecular Characterization of NSCLC in FFPE and cfDNA Samples

Samples from approximately 15% of the patients (n = 13) were chosen for validation of cobas^®^ EGFR Mutation Test v2 (Roche Diagnostics, Mannheim, Germany) results. Real-time PCR assays with a Pan Lung Cancer PCR Panel (Amoy Diagnostics, Xiamen, China) were used to detect DNA-based mutations and mRNA-based fusions in driver genes. Formalin-fixed paraffin-embedded (FFPE) tumor tissues and cfDNA samples were used to detect 167 hotspot variants in EGFR/ALK/ROS1/KRAS/BRAF/HER2/RET/MET/NTRK1/NTRK2/NTRK3 genes. Genomic DNA and total RNA were automatically extracted from FFPE samples using the MagCore Genomic DNA FFPE One-Step and MagCore Total RNA FFPE One-Step kits, respectively, following the manufacturer’s protocols (RBC Bioscience, New Taipei City, Taiwan). The DNA and RNA were eluted with 60 μL elution buffer and quantified using a Qubit dsDNA HS assay and Qubit RNA HS assay kit (Thermo Fisher Scientific, Waltham, MA, USA). cfDNA from plasma samples was extracted as described above for NGS analysis. Subsequently, samples were prepared following Amoy Dx guidelines for cfDNA, genomic DNA and total RNA.

### 2.6. Library Preparation and Sequencing

The QIAseq Human Lung Cancer Panel (DHS-005Z; QIAGEN, Hilden, Germany) was chosen for the analysis of discordant cases as it detects SNVs, CNVs and small indels of 72 genes relevant in lung cancer. Libraries were prepared following QIAGEN guidelines for cfDNA and the protocol was as follows. Briefly, 10–20 ng of cfDNA was enzymatically fragmented and end-repaired in a 25 μL reaction volume containing 2.5 μL 10 × fragmentation buffer, 1.25 μL FG solution and 5 μL fragmentation enzyme mix. The reaction was carried out at 4 °C for 1 min, 32 °C for 14 min and 72 °C for 30 min. Next, 10 μL 5 × ligation buffer, 5 μL DNA ligase, 7.2 μL ligation solution and 0.5 μL barcoded adapters were added to 2.3 μL water to reach a reaction volume of 50 μL. Reaction tubes were then incubated at 20 °C for 15 min. To ensure complete removal of free barcoded adapters, each reaction was purified twice using 112 μL and 70 μL of QIAseq beads, respectively. The purified cfDNA was then mixed in a 20 μL reaction volume with 5 μL of QIAseq Human Lung DNA Panel primers, 0.8 μL IL-Forward primer, 4 μL 5× TEPCR buffer and 0.8 μL HotStarTaq DNA polymerase. The target enrichment PCR protocol used was: 95 °C for 13 min; 98 °C for 2 min; six cycles of 98 °C for 15 s and 65 °C for 15 min; and 72 °C for 5 min. Each reaction was cleaned again once using 112 μL QIAseq beads. Enriched DNA was then combined with 0.8 μL IL-Universal primers, 0.8 μL IL-Index primers, 4 μL 5 × UPCR buffer and 1 μL HotStarTaq DNA polymerase in a volume of 20 μL. The universal PCR conditions were as follows: 95 °C for 13 min; 98 °C for 2 min; 22 cycles of 98 °C for 15 s and 60 °C for 2 min; and 72 °C for 5 min. The DNA library was then purified once with 108 μL QIAseq beads, resuspended in 40 μL and quantified using a Qubit dsDNA HS assay kit (Thermo Fisher Scientific, Waltham, MA, USA).

The Agilent 2100 Bioanalyzer High Sensitivity DNA kit (Agilent technologies, Santa Clara, CA, USA) was used to evaluate the quality, fragment size and concentration of purified libraries. Non-specific peaks were removed by performing additional clean-up step(s) with QIAseq magnetic beads. The libraries were normalized to 4 nM before pooling and further denatured and diluted to 8 pM for sequencing on an Illumina Miseq platform (paired-end, 2 × 300 bp) according to the manufacturer’s instructions (Illumina, San Diego, CA, USA).

### 2.7. Variant Calling and Data Analysis

The QIAseq Targeted DNA Panel Analysis software (CLC Genomics Workbench version 21; QIAGEN, Hilden, Germany) and QIAGEN Clinical Insight Interpret Translational (QCI, version 8.1.20210827; https://digitalinsights.qiagen.com/, accessed on 7 April 2022; QIAGEN, Hilden, Germany) were used to analyze the sequencing data and to generate variant reports. Only variants passing filter recommendations by the QIAGEN bioinformatics pipeline were used for subsequent analysis. Considered variants had at least 500X molecular tag coverage and an allelic fraction equal or greater than 1%. Reads were aligned to reference genome hg19. All the variants identified were verified using the Integrative Genomics Viewer (IGV) (https://www.broadinstitute.org/igv, accessed on 18 May 2022; Broad Institute, MA, USA). Somatic variants were distinguished from population polymorphisms by referencing the gnomAD database (http://gnomad.broadinstitute.org/, accessed on 20 May 2022; Broad Institute, MA, USA). Clinical significance evidence and implications of QCI-reported variants were contrasted with annotations in public archives: ClinVar (https://www.ncbi.nlm.nih.gov/clinvar/, accessed on 20 May 2022; Bethesda, MD, USA), Catalogues of Somatic Mutations in Cancer (COSMIC, http://cancer.sanger.ac.uk/cosmic, accessed on 20 May 2022; Sanger Institute Catalogue of Somatic Mutations in Cancer, Cambridgeshire, UK), VarSome (https://varsome.com/, accessed on 20 May 2022; Saphetor SA, Lausanne, Switzerland) and Clinical Interpretations of Variants in Cancer (CiVic, https://civic.genome.wustl.edu/home, accessed on 20 May 2022; McDonnell Genome Institute, St. Louis, MO, USA).

### 2.8. Statistical Analysis

Statistical analyses were performed using the IBM SPSS^®^ Statistics v27 program (SPSS Inc., Chicago, IL, USA). Descriptive statistics have been used for characterizing the clinical and pathological data of the patients in the study. Venn diagrams were used for comparisons of the different mutations detected. The strength and direction of correlation were calculated using the coefficient phi (φ). The most relevant mutations (Ex19Del, L858R and T790M) were chosen as dichotomous nominal variables (absence/presence). The Kaplan–Meier model was used to calculate the cumulative survival comparing the prognostic significance between EGFR-mutated patients, T790M-positive patients and the wild type genotype, as well as the survival curve during TKI treatment time. Cumulative survival rates were compared between subgroups by the Breslow test. Statistical significance was determined at α-limit = 5%.

## 3. Results

### 3.1. EGFR Mutations Detected in Tissue and Plasma Samples

#### 3.1.1. Characteristics of Patient Cohort and Presence of Initial EGFR Mutation

We have studied 158 cfDNA samples belonging to 86 NSCLC patients analyzed by the Pathological Anatomy Service of the University Hospital Complex of A Coruña between May 2016 and February 2022. All of them were suitable to be treated with TKIs, so plasma samples underwent analysis for detection of EGFR mutations. Clinical data such as gender, age, tumor histology, smoking habits, disease stage and cancer treatment were obtained from the medical records and are summarized in Table 1 and Table S1 (Supplementary Materials). Most of the patients presented an advanced stage (III–IV) of the disease at the time of the analysis and sixty of them had already died when writing this article.

**Figure 1:**
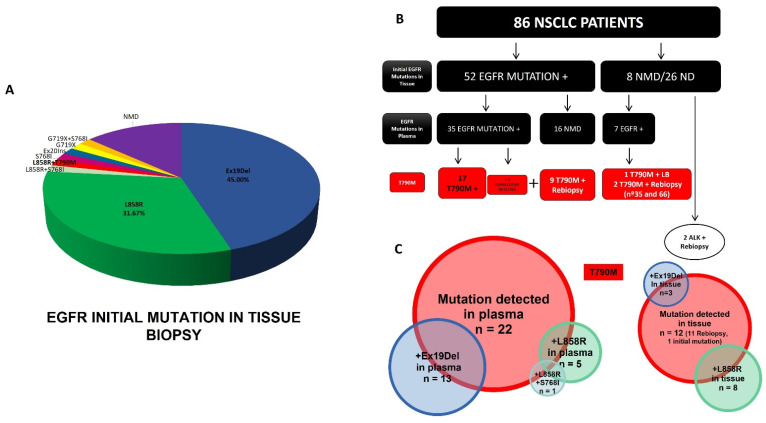
Summary of EGFR mutations analyzed. (**A**) EGFR mutations detected in initial tumor biopsies. (**B**) EGFR mutation analysis of patient cohort. (**C**) T790M mutation detection in plasma and tissue biopsies.

In the first instance, we performed a retrospective study of the initial EGFR mutations of the patient cohort detected in tissue biopsies. All patients presented results of previous EGFR mutational studies except 26 patients where EGFR mutational status could not be determined (ND). We considered as previous studies those carried out before, simultaneously with or a maximum of 30 days later with respect to the liquid biopsy analysis. It is assumed that in this 30-day interval the EGFR mutational status does not vary despite the possible targeted therapy that may be received at this time. All tissue biopsies performed more than 30 days after an initial biopsy (in lymph nodes or other locations) were considered tissue rebiopsies and classified as such in the present study. Results of EGFR mutations in tissue biopsies are shown in Figure 1A and Table 2. In summary, 76.67% of the patients harbored the most frequent sensitivity mutations (exon 19 deletion and L858R). In 13.33%, no mutation was detected (NMD) and the rest of the patients presented less frequent mutations such as S768I, G719X, Ex20Ins, with two cases harboring S768I + G719X and S768I + L858R. In only one case, we found a patient (no. 54) with the T790M resistance mutation in an initial tissue biopsy (L858R + T790M), a patient whose disease debuted in stage IV with metastases. These are intriguing data since this initial biopsy is performed for the staging and classification of patients before treatment.

#### 3.1.2. EGFR Mutations Detected in Plasma Samples

All patients included in our study (n = 86) underwent EGFR mutational analysis in plasma (158 cfDNA total analysis). The results are shown in Figure 1B and Table 2. More than half of patients were subject to only one analysis (n = 50); the rest of them were monitored two (n = 16), three (n = 11), four (n = 6), five (n = 1, no. 63), six (n = 1, no. 7) and even seven times (n = 1, no. 41). In all the analyses performed, 32.56% of them presented a non-mutated genotype (wild type), both in tissue and plasma. In 67.44% of the samples (n = 58 patients), several EGFR mutations were detected. These results are described in Table 2.

In five patients (no. 28, 66, 74, 84 and 85), liquid biopsy was useful for detecting TKI-sensitive alterations that could not be detected in tissular biopsy. Regarding T790M mutation (Table 2, marked in red), of the 58 patients in whom some EGFR mutations were detected, 31.03% (n = 18) harbored the resistance alteration. In nine cases, the mutation was not detectable in the first analysis but was positive in subsequent ones. These data showed one of the limitations of the technique, the possibility of false negative values. Furthermore, as shown in Figure 1C and Table 2, in plasma, T790M was associated with exon 19 deletion 13 times in 11 patients, five times with L858R (also associated with the S768I mutation in no. 20) and only four times in isolation (no. 54 and no. 81). Patient no. 54 was also the only case in which this alteration, accompanied by L858R, was detected in initial tissue biopsy (Table 2, marked in yellow).

Only three patients (no. 23, 53 and 54) had positive results for the presence of T790M mutation in more than one plasma determination. As can be seen in Table S2, all of them were patients with disease progression after osimertinib treatment who subsequently underwent different therapeutic approaches due to the development of the malignancy. These different approaches, as well as the limitations inherent to the technique, make it difficult to infer a direct relationship between the results of the analysis and the targeted treatment.

#### 3.1.3. Patients Undergoing Rebiopsy

Furthermore, seventeen patients recorded in Table 2 underwent tissue rebiopsy at least 30 days after plasma analysis. The T790M mutation was detected in 11 of these cases, 10 of them as a de novo mutation, not detected in prior tissue or plasma determinations; while the ninth patient (no. 54) already presented this alteration in previous analysis. Thirty-six per cent of the cases (36.36%) had previously presented exon 19 deletion; the remaining 63.64%, the L858R alteration. All these T790M-positive patients had a recent negative liquid biopsy, except patient no. 54, which indicated the need to search for diagnostic alternatives when the progression of the disease indicates one pathway, and the sensitivity of the technique is not sufficient.

#### 3.1.4. Correlation between Mutations

For calculating the degree of association between the mutations of the cohort that we considered most relevant for our study (Ex19Del, L858R and T790M alterations) in initial sample (IM), liquid biopsy (LB) and/or rebiopsy (ReBx), we used the *phi* correlation coefficient (φ). Taking these mutations as dichotomous nominal variables (absence/presence), 2 × 2 contingency tables were made with all the possible combinations in all samples. Statistical significance was determined at α = 5% and the results are collected in Table 3.

A statistically significant, low and inversely proportional relationship was found between Ex19Del and L858R: in the initial Ex19Del and L858R mutations (φ: −0.385; *p* ≤ 0.001); between Ex19Del IM and L858R LB (φ: −0.385; *p* ≤ 0.001); between Ex19Del IM and L858R ReBx (φ: −0.217; *p* ≤ 0.05); between L858R IM and Ex19Del LB (φ: −0.292; *p* ≤ 0.01) and also Ex19Del LB and L858R LB (φ: −0.292; *p* ≤ 0.01). These data support the fact that these mutations are mutually exclusive [22,23]. Moreover, the individual relationship between the L858R and Ex19Del variation in primary tumor/rebiopsy and plasma was statistically significant, very strong and directly proportional (φ: 0.811; *p* ≤ 0.001/φ: 0.470; *p* ≤ 0.001 and φ: 0.637; *p* ≤ 0.001/φ: 0.308; *p* ≤ 0.01, respectively), demonstrating that in most cases the TKI-sensitivity mutation was not lost.

Regarding T790M mutation, a statistically significant relationship was found, with a weak and directly proportional association between the T790M resistance mutation in plasma and the deletion of exon 19, both in initial biopsy (φ: 0.329; *p* ≤ 0.01) and in plasma (φ: 0.438; *p* ≤ 0.001). With respect to rebiopsy determinations, T790M presence was strongly associated with L858R in initial mutation, plasma and rebiopsy (φ: 0.300; *p* ≤ 0.01; φ: 0.216; *p* ≤ 0.05 and φ: 0.633; *p* ≤ 0.001, respectively), and to Ex19Del mutation in ReBx (φ: 0.328; *p* ≤ 0.01).

### 3.2. Liquid Biopsy as a Disease Monitor. Response to TKIs

The 48.84% of the patients (n = 42) who presented an alteration in the initial tissue underwent subsequent liquid biopsies more than 30 days apart. Forty-one of these patients received EGFR-targeted TKIs during this period (97.62%). Table 4 shows the variations in mutational status as well as the targeted treatment during the time from the initial mutation to the first and subsequent liquid biopsies.

The mean time between the initial mutation and the first liquid biopsy screening was 17.7 months. T790M resistance mutation was detected only in seven patients during that time (16.67%). The remaining patients needed more than one LB to detect resistance (16.67%, n = 7), died within a few months with negative determinations (average of 4.2 months; 45.24%; n = 19) or T790M was detected by other methods (rebiopsy; 11.90%; n = 5). Four patients were negative and were still alive during the study (9.52%).

Twenty-four patients (57.14%) received third generation TKIs. In thirteen of them, the resistance mutation T790M was previously detected in liquid biopsy (n = 6 in the first biopsy, n = 7 in the subsequent ones). In five patients, T790M was only detected in rebiopsy, with liquid biopsies being negative. In the remaining six patients, treatment with third generation TKIs was started from the time of diagnosis, due to inclusion in clinical trials or for other clinical indications.

Our results also showed the efficacy of treatment with classical TKIs in concordance with the literature [24,25]. Seventeen patients received only first and second generation treatment. Among them, 88.23% (15 patients) died a mean of 4.1 months since the last EGFR determination. Of the 24 patients who received third generation treatment, 66.67% (16 patients) were dead at the moment of writing this article with a mean of 14.8 months since the last liquid biopsy.

### 3.3. Prognostic Significance of the T790M EGFR Mutation and TKI Treatment

Regarding our last results, we calculated the median total survival of the disease by comparing those patients who had presented some EGFR mutation and specifically T790M mutation throughout the disease with those who had not presented such alterations. We covered a range of 10 years (120 months), considering that 95.3% of our study subjects were diagnosed in stage IV or III and overall survival rates for five years range from 5–10% [26,27]. For each patient, time from the moment of diagnosis of the disease to the moment of exitus or date of survival analysis was calculated. All patients with the T790M resistance mutation were treated with different TKIs. Most of them, except one (no. 15), were treated with third generation TKIs (see Supplementary Table S1).

Fifty-eight of them (67.4%) presented some EGFR mutation at some point in the progression of their disease. Regarding T790M alteration, 28 patients harbored a resistance mutation (32.6%) while in the remaining 67.4% (n = 58), no such alteration was detected. Until the end of follow-up, only 22.4% of patients with mutated EGFR (n = 13) were still alive, compared to 17.9% of the T790M-positive patients. Global survival was 20.9%, while wild type group survival was 17.9% (n = 5), and T790M-negative group survival was 22.4% (n = 13; Table 5).

The median overall survival of the patients in our study with a follow-up of 120 months was 38.0 months (95% CI: 28.6–47.5). In each group, the median survival in months of patients with mutated EGFR was 44.7 (95% CI: 31.1–58.2) in contrast to that of patients without any alteration, which was 14.8 (95% CI: 2.4–27.1). T790M-positive patients presented a median overall survival of 44.7 months (95% CI: 20.7–68.6), the same as the EGFR-mutated group. T790M-negative patients showed a median survival of 27.2 months (95% CI: 15.6–38.8) instead.

**Figure 2:**
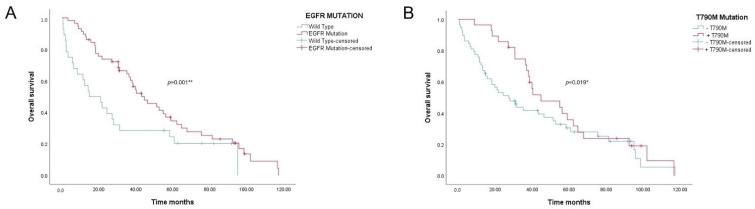
(**A**) Overall survival curve for patients harboring EGFR mutations vs. wild type patients; *p* value = 0.001. (**B**) Overall survival curve for patients harboring T790M mutations vs. T790M-negative patients; *p* value = 0.019. Asterisks *, **, denotes significant at a *p*-value < 0.05, 0.01, respectively.

To assess whether these differences were statistically significant, survival analyses were performed using the Breslow test. Results are represented in the Kaplan–Meier curves in Figure 2. The EGFR mutation curve yielded a *p* value = 0.001. Forty months after the diagnosis of NSCLC, the EGFR-positive group had a survival rate of more than 50%, in contrast to the wild type group, in which this percentage drops to less than 30% survival (Figure 2A). The resistance mutation curve also showed significant differences between groups (*p* = 0.019, Figure 2B). Survival of the group with mutated EGFR was significantly higher, since these patients underwent TKI treatments with better OS rates. However, in both cases, after 60 months, survival rates tended to equalize, probably due to the appearance of new resistance mutations or other associated processes.

**Figure 3:**
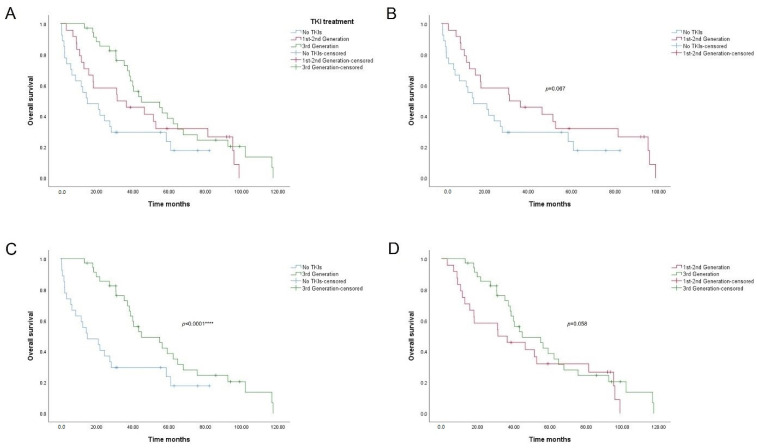
Overall survival curves for patients undergoing TKI treatment. (**A**) Overall comparison between treatments. (**B**) No TKIs vs. 1st–2nd generation (*p* value = 0.067). (**C**) No TKIs vs. 3rd generation (*p* value < 0.0001 ****). (**D**) 1st–2nd generation vs. 3rd generation (*p* value = 0.058). Asterisks **** denotes significant at a *p*-value < 0.0001, respectively.

Considering these results, we wanted to verify if there were significant differences in survival according to the type of TKI treatment (Table 6; Figure 3). The median overall survival in months of patients not treated with TKIs and treated with first–second generation TKIs was 14.8 months (95% CI: 0.4–29.6) and 31.2 months (95% CI: 0.0–64.2), respectively. Overall survival of patients treated with third generation TKIs was significantly higher, 44.7 months (95% CI: 24.7–64.7).

Regarding Kaplan–Meyer curves, we found significant differences between the patients who were not treated with TKIs and those who were treated with third generation drugs (Figure 3C, *p* value < 0.0001). The latter had much higher survival rates (nearly double) up to 40 months. From that time on, survival in the third generation group dropped below 20% after 80 months regarding mutation analysis (Figure 2). No differences were found between the other pairs of groups (Figure 3B, *p* value = 0.067; Figure 3D, *p* value = 0.058), although median overall survival data were different.

### 3.4. Validation of EGFR Results in Tissue and Plasma Samples

Samples from approximately 15% of the patients (n = 13) were chosen for validation of cobas^®^ EGFR Mutation Test v2 (Roche Diagnostics, Mannheim, Germany) results. Real-time PCR assays with a Pan Lung Cancer PCR Panel (Amoy Diagnostics, Xiamen, China, CE-IVD) were used to detect DNA-based mutations and mRNA-based fusions in driver genes. Six samples of cfDNA and seven tissue rebiopsies were randomly selected. Comparative results are shown in Table 7.

Data showed a complete concordance between the two methods for detection of EGFR mutations. Furthermore, two KRAS driver mutations in exon 2 were also detected by Pan Lung Cancer Panel in patients no. 7 (G12A/V/R/G13C) and no. 65 (G12C). Both patients harbored an Ex19Del sensitivity mutation on initial biopsy and T790M was not detected. KRAS mutation in initial biopsy in patient no. 65 was not detected either. No sample was available to detect other mutations in initial biopsy of no. 7. Both patients died of disease evolution after treatment with TKIs. The existence of other concomitant secondary driver mutations would explain the progression of the disease after resistance to targeted therapies in these cases [28,29]. No fusions, rearrangements or other DNA alterations were detected in the rest of the samples.

### 3.5. NGS Analysis of Patients with Discordant Progression

**Figure 4:**
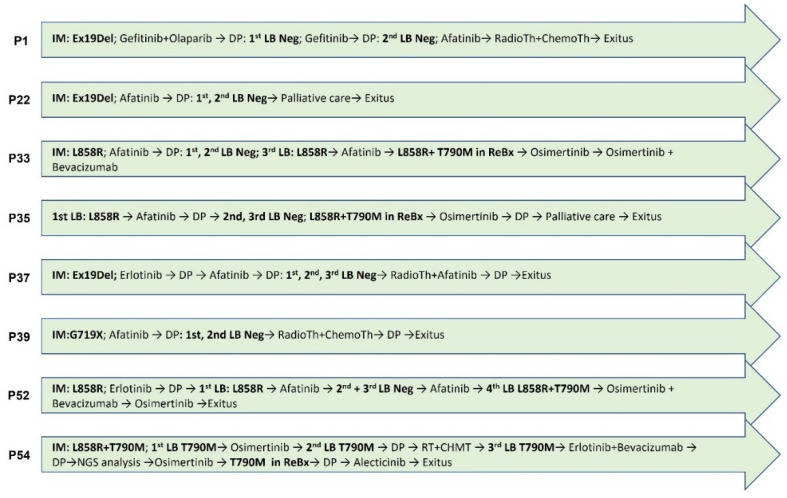
Disease progression and treatments of patients undergoing NGS analysis. All patients experienced disease progression after TKI treatment. IM: initial mutation. DP: disease progression. LB: liquid biopsy. ReBx: rebiopsy.

Finally, we found patients with discordant progression whose clinical signs we were not able to analyze with the available data. Thus, eight plasma samples belonging to eight patients with conflicting clinical evolution who did not respond to treatment and/or in which T790M TKI resistance mutation was not detected were analyzed by NGS Human Lung Cancer Panel (QIAGEN, Hilden, Germany). In six of them, T790M resistance mutation was not detected in all liquid biopsies either. All of them harbored sensitive mutations in initial biopsy so were subject to be treated by TKIs. Disease progression and treatments of the eight subjects are collected in Figure 4.

**Figure 5:**
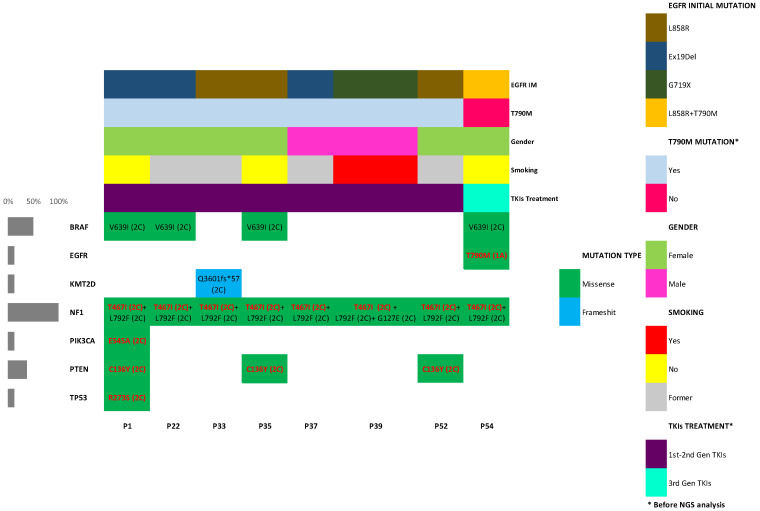
Commutation plot of filtered pathogenic and likely pathogenic variants detected in cfDNA of patients with discordant progression. Pathogenic alterations are highlighted in red font. Tier classification is stated in brackets. All patients harbored T467I + L792F mutations in tumor suppressor NF1 gene. Asterisk denotes that T790M mutation status and TKI treatment received were considered just before NGS analysis.

All filtered variants identified are summarized in Supplementary Tables S3–S10. Variant calling and data analysis protocols are explained above. Clinically relevant pathogenic or likely pathogenic variants identified by the QCI program are collected in Figure 5. A commutation plot showed the identified alterations, their potential actionability based on tier classification (according to the 2015 professional guidelines from the American College of Medical Genetics and Association for Molecular Pathology (ACMG/AMP) [30] and the 2017 guidelines from the Association for Molecular Pathology, American Society of Clinical Oncology and College of American Pathologists (AMP/ASCO/CAP) [31]) and their pathogenicity based on 2015 ACMG/AMP guidelines [30]. All variants considered pathogenic or likely pathogenic based on QCI predictions were considered tier 2C (FDA-approved therapies for different tumor types or investigational therapies with multiple small published studies with some consensus), except T790M, considered 1A (FDA-approved therapy included in professional guidelines).

Patients no. 1, 22, 33, 37 and 39 were treated by first and second generation TKIs. Resistance mutations were not detected in plasma samples or even rebiopsy in patient no. 22. Plasma sample analysis by NGS was carried out in the case of no. 1 after treatment with gefitinib + olaparib when disease progressed. In patients no. 22, no. 33, no. 37 and no. 39, a sample was collected after treatment with afatinib in all cases. Massive sequencing data did not show relevant EGFR alterations in any of the cases. Only in patient no. 1 was more than one pathologically significant alteration found in NF1 (c.1400C > T; p.T467I and c.1642-3C > G), PIK3CA (c.1634A > C; p.E545A), PTEN (c.407G > A; p.C136Y) and TP53 (c.817C > A; p.R273S) genes. The mutation found in the PIK3CA gene in exon 9 (c.1634A > C; p.E545A) was particularly significant since it is related to a worse prognosis of the disease [32]. The rest of the patients only presented a mutation with pathogenic significance in the NF1 gene (c.1400C > T; p.T467I).

T790M EGFR resistance mutations were not detected in prior liquid biopsies by PCR in patients no. 35 and no. 52 before NGS analysis. They both underwent a tumor rebiopsy (no. 35) and subsequent liquid biopsy (no. 52) five and three months, respectively, after NGS–plasma analysis, and a double mutation of L858R + T790M was detected. A sample after afatinib + erlotinib treatment was analyzed by Human Lung Cancer Panel in patient no. 35 and data showed no EGFR resistance mutations. NF1 (c.1400C > T; p.T467I) and PTEN (c.407G > A; p.C136Y) alterations were found as the most relevant variants. In patient no. 52 after afatinib treatment, data showed the same results, not EGFR alterations, but the same variants in NF1 (c.1400C > T; p.T467I) and PTEN (c.407G > A; p.C136Y) genes.

Finally, patient no. 54 harbored T790M mutation in all biopsies (tissue and plasma samples) analyzed. She was the only patient who had the resistance mutation in the initial biopsy before any treatment. She was treated with osimertinib and other targeted therapies but suffered clinical worsening. A plasma sample was analyzed after third generation TKI treatment. Analysis revealed only the presence of T790M mutation and NF1 modification (c.1400C > T; p.T467I) as pathogenic variants.

An NF1 (c.1400C > T; p.T467I) variant was found in all samples studied with high allelic frequencies (from 5.23% to 22.0%, Tables S3–S10). The rest of the relevant variants that were detected were likely pathogenic alterations in the BRAF and KMT2D genes and other NF1 alterations. One of these variants (c.2374C > T; p.L792F) was also found in all patients but the significance of these mutations in the NF1 tumor suppressor gene in non-small cell lung cancer has not been demonstrated [33].

## 4. Discussion

First and second generation of EGFR TKIs were effective first-line treatments for EGFR mutant NSCLCs [34,35]. About 75% of patients with this type of neoplasia had a good outcome with these therapies, but these responses usually were not permanent, and patients developed resistance after several months of treatment [22,35]. Several mechanisms of acquired resistance have been described, including the development of the “gatekeeper” point mutation that leads to Thr to Met substitution at residue 790 (T790M) of the EGFR gene. T790M is the most common resistance mechanism and can be observed in over 50% of patients [36,37]. This mutation increases the ATP binding affinity of the oncogenic activating mutants and prevents TKIs from effectively inhibiting EGFR. Third generation TKIs such as osimertinib carry an acceptor functional group to irreversibly alkylate a cysteine residue (C797) in the ATP binding site of EGFR, overcoming this problem [38]. More than 50% of patients harboring EGFR T790M respond well to these treatments and it also seems to improve first and second generation side effects [39]. However, despite these positive results, a new acquired resistance has been developed to these drugs. Mutations L718Q, L844V and C797S were identified from subjects with advanced lung cancer that had developed resistance to third generation TKIs, proving that these drug-resistance mutations are clinically relevant [17,40]. In these NSCLC patients, mechanisms observed in cancers with acquired resistance to first generation EGFR inhibitors have also been identified. These include phenotypic/histological changes such as epithelial to mesenchymal transition (EMT) or SCLC transformation, constitutive activation of the MAPK kinase pathway [18] or IGF1R bypass track signaling [41,42]. In addition, it is also important to take into account the intrinsic resistance to TKIs, which may be due to the pre-existence of concomitant mutations in driver genes or alterations in tumor suppressors. [43]. Most of the patients studied in the present work did not respond to TKI treatment, although 76.7% presented L858R and exon 19 deletion sensitivity mutations, the most frequent EGFR genomic alterations [44]. T790M appeared secondarily as a mechanism of resistance to treatment with tyrosine kinase inhibitors in a smaller percentage of cases (32.56%), most of the time associated with the deletion of exon 19. One hundred per cent of the patients in our study who became resistant and mutated T790M had received treatment with first or second generation TKIs. However, most of the patients did not present the alteration, thus opening several possible explanations for this resistance to therapy: intrinsic resistance, new unknown acquired resistance mechanisms, mutations not detectable by the test (RAS, MET, HER2 or EGFR amplification, mutations of PIK3CA or transformation to SCLC) or the non-negligible rate of false negatives in liquid biopsy compared to tissue biopsy [45]. Consequently, we see a limitation of this technique when it comes to understanding and managing possible secondary and tertiary resistance in these patients. This genomic heterogeneity requires a more exhaustive study, although it advocates the need for combination therapies that prevent or inhibit the appearance of these simultaneous resistance mechanisms [17,41]. At this level, the role of other techniques such as next generation sequencing (NGS) or digital droplet PCR (ddPCR) is of great importance, as they are able to overcome many of the limitations of liquid biopsy due to the wide range of mutational detection and high sensitivity [46]. In this situation, it is also important to highlight the role of rebiopsies, which offer very important additional information in that specific context. They are an invasive and challenging method, and their performance is not always possible, but the data they provide would complete the knowledge obtained from liquid biopsies [47]. However, as we showed with our results, cfDNA analysis can be a useful tool for monitoring disease. We can monitor variations in mutational status as well as the effectiveness of targeted treatment from the beginning through subsequent plasma analysis. Furthermore, liquid biopsy is a simple, cheap and available test, and with the support of rebiopsies, can offer a general landscape of the evolution of the disease [13].

Association studies in our cohort of patients also showed a statistically significant relationship between EGFR L858R and Ex19Del sensitivity mutations in initial tissue and their corresponding liquid biopsy. These data were relevant since recent studies have observed differences between NSCLC patients with exon 19 deletion and L858R mutation in terms of the efficacy of EGFR TKIs. These observations concluded that subjects harboring L858R mutation showed a significantly lower response than those with deletion of exon 19 [48]. Consequently, detecting these alterations in plasma not only allows initiation of targeted therapies, but also prediction of the response to them.

Regarding prognostic significance of T790M mutation and TKI treatment, we observed that the presence of resistance mutation and associated targeted treatment significantly increased the OS rates in our cohort of patients. Median overall survival of patients treated with third generation TKIs and mutated T790M was much higher up to 40 months. OS rates tended to converge after 60 months, probably due to the appearance of new resistance mutations or other associated processes.

On the other hand, although T790M is considered as a resistance mutation, this alteration of exon 20 of EGFR may appear as an initial mutation (in our case, associated with L858R), so it does not necessarily originate in response to treatment with inhibitors from the first line. In fact, previous studies have found a frequency close to 17% in the Caucasian population with a baseline T790M mutation, associated with a sensitizing EGFR mutation, especially the deletion of exon 19 [49]. Based on this and the superior efficacy of osimertinib as a therapy against EGFR mutations (not only T790M), osimertinib is currently used as standard first-line treatment in patients with common mutations (Ex19Del, L858R) [38,50,51], without the prior step of using reversible inhibitors. There are already studies that support this decision by demonstrating a higher response rate and survival rate at 18 months [7,52]. These are data that correlate with our results.

In relation to the appearance of new resistance mutations, by randomly validating the results obtained of the detection of EGFR mutations, we observed the presence of two driver mutations in exon 2 of KRAS (G12X), which could help interpret bad outcomes of patients who harbored such alterations. In at least one case (G12C, no. 65), this driver mutation appeared secondarily after treatment with TKIs. EGFR and KRAS mutations in NSCLC were generally considered to be mutually exclusive. However, KRAS is mutated in ∼30% of NSCLC cases and concomitant mutations were found in an increasing number of patients [28,29]. Furthermore, it has also been identified as one of the mechanisms underlying resistance to TKIs in EGFR-mutated cases [53,54]. Recent studies showed that KRAS p.G12C mutation was detected in 1% of EGFR-positive NSCLC patients who progress with a first-line TKI [25], and last year the FDA approved sotorasib as the first inhibitor of KRAS in NSCLC that acts by blocking KRAS-G12C mutation [55]. Therefore, subsequent studies on the detection of new resistance mutations in other driver genes pre- and post-treatment seem required.

In this sense, we performed NGS analysis of available plasma samples from patients who had discordant disease progression, searching for new acquired mutations or unidentified intrinsic alterations that could have clinical significance. Our results showed pathogenic variants in tumor suppressor genes such as NF1, PTEN or TP53, but whose relevance in NSCLC has not yet been demonstrated and no literature was available. However, some reports showed that in adenocarcinomas and squamous cell lung carcinomas, TP53, RB1, ARID1A, CDKN2A, PIK3CA and NF1 genes were significantly mutated in both tumor types [56]. Other studies found that non-small cell lung tumors carried subclonal driver alterations in genes such as PIK3CA, NF1, KRAS, TP53 and NOTCH family members and some of these alterations occurred secondarily in evolution in more than 75% of the tumors [57]. PIK3CA and NF1 variants commonly appeared during disease development as well as mutations in genes that were involved in chromatin modification and DNA damage response and repair.

Surprisingly, our results showed that all patients harbored the same pathogenic mutation in the NF1 gene (p.T467I). NF1 p.T467I is a missense alteration predicted to be deleterious by computational methods and may impact gene function. NF1 T467I is prior to the GAP-related domain of the neurofibromin protein and has not been functionally characterized so its effect on protein function is still unknown [58]. However, it has been associated with ALK-fused spitzoid neoplasms [59], melanoma [60] or liver neuroendocrine tumors [61], among others. No relevant data were found in the literature related to lung cancer. We detected the variant in good percentages of allelic frequency and high read depth coverage in all patients, so we dismissed an artifact issue. Moreover, other pathogenic NF1 alterations were detected, such as the p.L792F variant, in all patients analyzed, but, although NF1 variants occur in lung cancer, their clinical significance is not well documented [33,62]. It has been reported that TKI resistance was associated with silencing and reduced expression of NF1 responding to MEK inhibitors [63], so our preliminary data could indicate that NF1 alterations, and specifically the p.T467I pathogenic variant, may act as a predictor of resistance. Studies with a larger number of patients, and with rigorous follow-up analysis, are necessary to ensure this fact. Moreover, the origin of these alterations (intrinsic or acquired) must also be analyzed. Our data were found in patients with unknown resistance to treatment, and although the number of patients was low and data were precursory, in the evolution of EGFR-mutated lung cancer, NF1 could be playing an unknown role in disease progress and resistance to targeted therapies.

## 5. Conclusions

After the results of our study, we can conclude that despite the lack of standardization of the technique and its inherent limitations, the implementation of liquid biopsy as a diagnostic method in NSCLC is a very useful tool for monitoring the evolution of the disease and the resistance to 3rd generation TKIs. Nevertheless, NSCLC is a complex disorder, so it is still fundamental to study the initial biopsy, as well as rebiopsies whenever feasible, in order to have the most complete landscape possible. Accordingly, it is not only important to detect T790M resistance mutation, but also other concomitant mutations in driver genes that could be influencing intrinsic and/or acquired resistance to targeted therapies and disease progression. In this sense, we have discovered that alterations in the NFI tumor suppressor gene could be playing a role and exerting a function in lung cancer that is unexplored to date.

## Figures and Tables

**Table 1:** Baseline characteristics of the patients.

Variables	No.	%	Variables	No.	%
Patients	86		Pathological stage		
Male	35	40.70	I	2	2.33
Female	51	59.30	III	6	6.98
Age (yr)			IV	77	89.53
Mean (range)	66.45 (36–89)		ND	1	1.16
Histology			Smoking		
Adenocarcinoma	74	86.05	No	38	44.19
Squamous cell carcinoma	3	3.49	Former smoker	28	32.56
Other NSCLC	9	10.47	Yes	16	18.60
			ND	4	4.65

**Table 2:** Detected mutations of the EGFR gene in tissue and liquid biopsy/ies in the patient cohort. Detection of T790M mutation are highlighted in yellow, green and red in initial tissue biopsy, rebiopsy and liquid biopsy/ies, respectively.

No.	Initial EGFR Mutation	Rebiopsy 1	Rebiopsy 2	LB1	LB2	LB3	LB4	LB5	LB6	LB7	No.	Initial EGFR Mutation	Rebiopsy 1	LB1	LB2	LB3	LB4	LB5
1	Ex19Del			NMD	NMD						43	Ex19Del		Ex19Del				
5	Ex19Del	T790M		NMD							44	L858R		NMD	L858R	NMD	NMD	
6	Ex19Del			Ex19Del	Ex19Del T790M						46	L858R		L858R				
7	Ex19Del	NMD	NMD	NMD	NMD	NMD	NMD	Ex19Del	Ex19Del		47	S768I		NMD	NMD			
8	Ex19Del			Ex19Del	Ex19Del T790M						50	Ex19Del		Ex19Del T790M				
9	Ex19Del			NMD	Ex19Del T790M						52	L858R		L858R	NMD	NMD	L858R T790M	
10	L858R			L858R							53	Ex19Del		NMD	Ex19Del T790M	Ex19Del T790M		
11	L858R			NMD	L858R	L858R					54	L858R T790M	L858R T790M	T790M	T790M	T790M		
13	Ex19Del			NMD	Ex19Del	Ex19Del T790M					55	L858R	L858R T790M	NMD	L858R			
15	Ex19Del			Ex19Del T790M							56	L858R		L858R	L858R			
20	S768I, L858R			S768I	NMD	S768I L858R T790M					59	L858R	L858R T790M	L858R	INV	L858R	L858R	
22	Ex19Del	Ex19Del		NMD	NMD	NMD					60	L858R		NMD	L858R T790M			
23	Ex19Del			NMD	Ex19Del T790M	Ex19Del	Ex19Del T790M				62	Ex19Del		NMD				
24	Ex20Ins			NMD							63	Ex19Del		NMD	NMD	NMD	NMD	NMD
27	L858R			L858R T790M							65	Ex19Del	NMD	NMD	NMD			
28	ND	INV	Ex19Del	Ex19Del							66	ND	Ex19Del T790M	Ex19Del	Ex19Del			
30	L858R	L858R		L858R	L858R	INV	INV				67	L858R		NMD				
31	L858R			L858R							69	G719X S768I		NMD				
32	Ex19Del			Ex19Del T790M							70	Ex19Del	Ex19Del T790M	Ex19Del				
33	L858R	L858R T790M		NMD	NMD	L858R					73	Ex19Del		NMD				
34	Ex19Del			Ex19Del T790M							74	ND	Ex19Del	NMD				
35	ND	L858R T790M		L858R	NMD	NMD					79	Ex19Del		NMD	NMD	NMD		
36	Ex19Del			Ex19Del							80	L858R	L858R T790M	L858R	NMD	NMD		
37	Ex19Del			NMD	NMD	NMD					81	Ex19Del		T790M				
38	L858R			L858R							82	L858R		NMD				
39	G719X			NMD	NMD						83	Ex19Del		Ex19Del T790M				
40	L858R	L858R T790M	NMD	L858R							84	ND	L858R	L858R				
41	Ex19Del		NMD	NMD	INV	NMD	NMD	NMD	NMD	NMD	85	ND		L858R				
42	Ex19Del	Ex19Del T790M	Ex19Del	NMD	NMD						86	L858R		L858R T790M				

**Table 3:** The 2 × 2 contingency tables, correlation coefficient phi (φ) and significance of different combinations of initial mutation (IM) and mutations in liquid biopsy (LB) and rebiopsy (ReBx). ^ns^, *, **, ***, not significant, significant at a *p*-value < 0.05, 0.01 or 0.001, respectively.

	Ex19Del IM	L858R IM	Ex19Del LB	L858R LB	T790M LB	Ex19Del ReBx	L858R ReBx	T790M ReBx
Ex19Del IM		φ: −0.385 ***	φ: 0.637 ***	φ: −0.385 ***	φ: 0.329 **	φ: 0.110 ^ns^	φ: −0.217 *	φ: −0.011 ^ns^
L858R IM			φ: −0.292 **	φ: 0.811 ***	φ: 0.107 ^ns^	φ: −0.156 ^ns^	φ: 0.470 ***	φ: 0.300 **
Ex19Del LB				φ: −0.292 **	φ: 0.438 ***	φ: 0.308 **	φ: −0.165 ^ns^	φ: 0.081 ^ns^
L858R LB					φ: 0.107 ^ns^	φ: −0.156 ^ns^	φ: 0.470 ***	φ: 0.216 *
T790M LB						φ: −0.191 ^ns^	φ: −0.066 ^ns^	φ: −0.097 ^ns^
Ex19Del ReBx							φ: −0.088 ^ns^	φ: 0.328 **
L858R ReBx								φ: 0.633 ***
T790M ReBx								

**Table 4:** Status mutation variation in patients who underwent a subsequent liquid biopsy more than 30 days after tissue biopsy. LBs: 0 = mutation disappeared; 1 = mutation remained; 2 = T790M mutation appeared (highlighted in red). TKI treatment: 0 = no treatment; 1 = 1st and/or 2nd generation TKIs; 2 = 3rd generation TKIs (highlighted in red); 3 = 1st/2nd + 3rd generation TKIs (highlighted in red).

No.	Months	1st LB	TKI Treat	Months	Subsequent LBs	TKI Treat	Rebiopsy	Exitus	No.	Months	1st LB	TKI Treat	Months	Subsequent LBs	TKI Treat	Rebiopsy	Exitus
1	37.2	0	1	4.2	0	1		5.3	44	4.9	0	1	50.7	1	1		
5	20.0	0	3				T790M	10.5	46	5.6	1	1					4.8
7	5.7	0	3	27.1	1	1	NMD	2.3	47	32.9	0	0	31	0	3		11.1
8	35.8	1	1	0.2	1	0		0.3	50	31.7	2	3					
9	14.9	0	1	0.2	2	2		22.4	52	17.1	0	1	21.4	2	3		37.2
10	1.8	1	1					1.0	53	21.8	0	1	32.5	2	2		3.6
11	4.0	0	1	5.6	1	1		0.6	56	8.6	1	2	2.2	1	2		1.4
13	15.7	0	1	14.3	2	3		38.1	59	26.2	1	1	3.0	1	2	L858R T790M	15.5
15	8.0	2	1					0.5	60	8.9	0	1	3.1	2	2		24.8
20	11.0	0	1	16.4	2	2		27.2	62	17.5	0	2					2.2
23	11.9	0	1	35.9	2	2		6.6	63	5.3	0	1	22.1	0	2		
27	11.7	2	3					14.5	65	37.4	0	1	10.2	0	1	NMD	5.0
30	9.5	1	1	5.0	1	0	L858R	0.7	67	13.9	0	1					3.7
31	4.87	1	2					2.0	69	11.8	0	1					21.8
32	10.0	2	3					7.8	70	9.2	1	2				Ex19Del T790M	12.2
33	38.4	1	1	19.9	1	2	L858R T790M		79	62.1	0	1	20.3	0	1		
36	9.2	1	1					3.3	80	57.1	1	1	0.5	0	2	L858R T790M	
37	20.5	0	1	0.5	0	0		7.9	81	21.0	2	3					
38	15.1	1	1					3.03	82	12.2	0	0					
39	2.0	0	1					6.2	83	16.7	2	3					13.8
43	2.0	1	1					1.3	86	18.3	2	2					

**Table 5:** Patients distributed according to the presence or absence of EGFR mutation (A), and T790M resistance mutation (B). Censored: no death.

A			Censored		B			Censored	
Mut Status	N Total	N Exitus	N	%	Mut Status	N Total	N Exitus	N	%
Wild Type	28	23	5	17.9%	T790M-	58	45	13	22.4%
EGFR+	58	45	13	22.4%	T790M+	28	23	5	17.9%
Global	86	68	18	20.9%	Global	86	68	18	20.9%

**Table 6:** Patients distributed according to TKI treatment: no treatment (Group A); treated with 1st−2nd generation TKIs (Group B); treated with 3rd generation TKIs (Group C). Censored: no death.

			Censored	
Mut Status	N Total	N Exitus	N	%
No TKIs	27	21	6	22.2%
1st–2nd Generation	24	20	4	16.7%
3rd Generation	35	27	8	22.9%
Global	86	68	18	20.9%

**Table 7:** Results of randomly selected samples for validation of EGFR data. Comparation between Pan Lung Cancer Panel and cobas EGFR Mutation Test. Asterisk (*) denotes KRAS results obtained by Pan Lung Cancer Panel but not analyzed by cobas^®^ EGFR Mutation Test.

No.	Type of Sample	Pan Lung Cancer Panel	cobas EGFR Mutation Test
		Mutation Detected	Mutation Detected
1	Liquid biopsy	Negative	Negative
5	Rebiopsy	T790M	T790M
7	Rebiopsy	KRAS G12A/V/R/G13C *	Negative
22	Liquid biopsy	Negative	Negative
30	Rebiopsy	L858R	L858R
31	Liquid biopsy	L858R	L858R
33	Rebiopsy	L858R T790M	L858R T790M
35	Liquid biopsy	Negative	Negative
37	Liquid biopsy	Negative	Negative
54	Rebiopsy	L858R T790M	L858R T790M
56	Liquid biopsy	Negative	Negative
65	Rebiopsy	KRAS G12C *	Negative
70	Rebiopsy	Ex19Del T790M	Ex19Del T790M

## Data Availability

The data presented in this study are available in the article and Supplementary Material. Any other related information or document not present in this study is available on request.

## Supplementary Materials

The following supporting information can be downloaded at: www.mdpi.com/article/10.3390/cancers14143323/s1: Table S1: Baseline characteristics of patients and EGFR mutations detected in tissue and plasma samples. Table S2: Disease progression and treatment of patients harboring T790M mutation in tissue and/or plasma samples. Table S3: Filtered variants detected in plasma in patient no. 1. Table S4: Filtered variants detected in plasma in patient no. 22. Table S5: Filtered variants detected in plasma in patient no. 33. Table S6: Filtered variants detected in plasma in patient no. 35. Table S7: Filtered variants detected in plasma in patient no. 37. Table S8: Filtered variants detected in plasma in patient no. 39. Table S9: Filtered variants detected in plasma in patient no. 52. Table S10: Filtered variants detected in plasma in patient no. 54.
